# Phenolic Acids from *Fructus Chebulae* *Immaturus* Alleviate Intestinal Ischemia-Reperfusion Injury in Mice through the PPARα/NF-κB Pathway

**DOI:** 10.3390/molecules27165227

**Published:** 2022-08-16

**Authors:** Junjie Liu, Bin Li, Jing Liu, Feng Qiu, Yunpeng Diao, Yuxin Lei, Jianjun Liu, Wei Zhang

**Affiliations:** 1 Department of Pharmacy, First Affiliated Hospital of Dalian Medical University, Dalian 116011, China; 2 College of Pharmacy, Dalian Medical University, Dalian 116044, China; 3 Dalian Anti-Infective Traditional Chinese Medicine Development Engineering Technology Research Center, Dalian 116044, China

**Keywords:** phenolic acids extract, *Fructus Chebulae Immaturus*, intestinal ischemia-reperfusion, PPARα/NF-κB-signaling pathway

## Abstract

Intestinal ischemia/reperfusion (II/R) injury is a common life-threatening complication with high morbidity and mortality. *Chebulae Fructus* *Immaturus*, the unripe fruit of *Terminalia chebula* Retz., also known as “Xiqingguo” or “Tibet Olive” in China, has been widely used in traditional Tibetan medicine throughout history. The phenolic acids’ extract of *Chebulae Fructus* *Immaturus* (XQG for short) has exhibited strong antioxidative, anti-inflammation, anti-apoptosis, and antibacterial activities. However, whether XQG can effectively ameliorate II/R injuries remains to be clarified. Our results showed that XQG could effectively alleviate II/R-induced intestinal morphological damage and intestinal barrier injury by decreasing the oxidative stress, inflammatory response, and cell death. Transcriptomic analysis further revealed that the main action mechanism of XQG protecting against II/R injury was involved in activating PPARα and inhibiting the NF-κB-signaling pathway. Our study suggests the potential usage of XQG as a new candidate to alleviate II/R injury.

## 1. Introduction

Intestinal ischemia/reperfusion (II/R) injury is a common life-threatening complication with high morbidity and mortality caused by the recovery of blood flow after intestinal ischemia injury [1], mainly manifested in mesenteric artery thrombosis, constricted intestinal obstruction, trauma, and intestinal transplantation [2,3,4]. The intestinal ischemia leads to a microvascular permeability increase, intestinal epithelial cell injury, and mucosal barrier dysfunction, while the reperfusion brings about inflammatory cell infiltration, excess oxidative products, and inflammatory cytokine release. Serious II/R injury can also result in severe local and systemic inflammation and multiple organ dysfunction syndrome [5]. As intestinal ischemia is rarely preventable, there is a substantial need for innovative therapeutic strategies to ameliorate II/R-induced injury [6].

The pathogenesis of II/R injury is complex and related to oxidative stress, inflammatory infiltration, epithelial cell death, and autophagy deficiency [7,8]. Multiple signaling pathways have been found to be correlated with II/R injuries, including the NF-κB, AMPK/Sirt1, and PPAR-signaling pathways [9,10,11]. The peroxisome proliferator-activated receptor-α (PPARα) is a ligand-activated nuclear receptor highly expressed in the liver, intestinal epithelial cells, and cardiac muscle cells [12]. PPARα will combine with the retinoid X receptor to form a heterodimer, activate the PPAR response element, and regulate the expression of the target genes involved in the regulation of fatty acid’s metabolism. It also decreases inflammation through inhibiting the NF-κB signaling-pathway by direct interaction with NF-κB or reducing the activated levels of NF-κB [13]. In addition to fatty acid metabolism and inflammation regulation, recent studies have shown that PPARα participates in various pathophysiological processes, including oxidative stress, apoptosis, and even tumorigenesis and cancer progression [14]. Growing evidence points to the specific PPARα activation by its agonist in the effective attenuation of I/R injury in the brain, heart, kidney, and liver, via inhibiting the inflammatory response, oxidative stress, and cell apoptosis [14]. PPARα has been considered as a potential drug target for the treatment of II/R injury.

*Terminalia chebula* Retz. is a medicinal plant of the Combretaceae family, which has been widely used in traditional Tibetan medicine throughout history. Both its ripe and unripe fruit have been used in folk medicine in Asia, with different clinical uses [15]. The unripe fruit, *Chebulae Fructus Immaturus*, also known as “Xiqingguo” or “Tibet Olive” in China, is a popular folk remedy for sore throat, pharyngitis, amygdalitis, and dysentery [16,17]. According to the *National Herbal Compendium*, it can treat diarrhea, stop bleeding, suppress lung infections, and resolve phlegm, and it is used in the treatment of chronic enteritis, chronic bronchitis, asthma, chronic laryngitis, ulcers, and hemafecia. Our group has recently shown that the main bioactive compounds of *Chebulae Fructus Immaturus* are phenolic acids, and we also investigated their biological activity, including the anti-oxidative, anti-inflammatory, anti-apoptotic, and antibacterial activity [18,19]. However, whether *Chebulae Fructus Immaturus* can effectively ameliorate the II/R injuries remains to be clarified. The aim of this study was to clarify the protective effect of *Chebulae Fructus Immaturus* on II/R injury and to demonstrate the underlying mechanism through transcriptomic analysis and bioinformatics tools.

## 2. Results

### 2.1. Determination of Total Phenolic Acids Content in Chebulae Fructus Immaturus Extract

**Figure 1:**
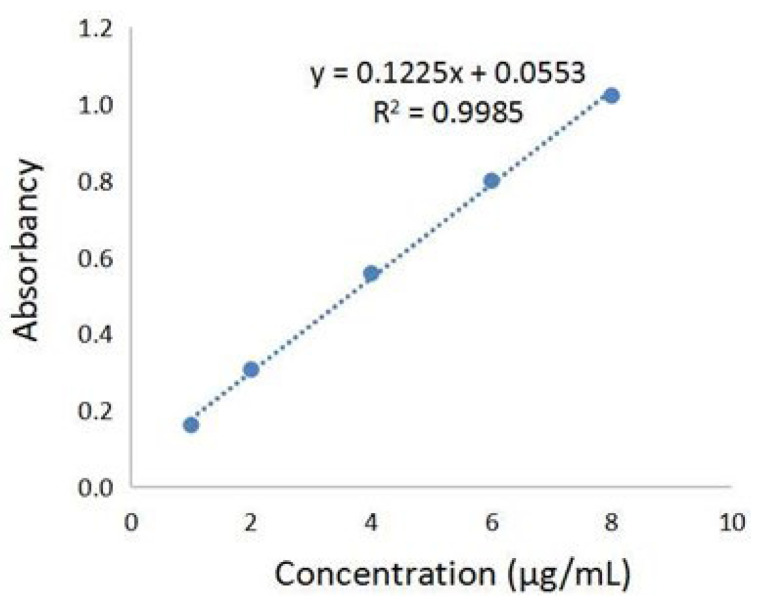
Gallic acid standard curve for the total phenolic acids content determination.

The phenolic acids’ extract from Fructus Chebulae Immaturus (referred to simply as XQG) were obtained, as previously described [18,19], and the total phenolic acids content was determined by the Folin–Ciocalteu method. The standard curve was established, as shown in Figure 1, with the absorbance value as the ordinate and the concentration of gallic acid reference solution as the ordinate. The results showed that there was a good linear relationship between the absorbance values of gallic acid at the concentration of 1~8 μg/mL. In this range, the linear regression equation was y = 0.1225x + 0.0553 (R^2^ = 0.9985), and the gallic acid equivalent of XQG was 111.58 ± 2.26 mg/g. Further, HPLC-MS/MS was applied to analyze the major components of XQG. As a result, 29 major components were identified, as shown in Figure S1 and Table S1 (Supplementary Materials). According to the base peak intensity, the 10 most abundant components are: Caffeic acid; Corilagin; Euphormisin M3; Chebuloside 2; Shikimic acid; Gallic acid; Chebulagic acid; Ellagic Acid; Dehydrodigallic Acid; and 3,4-Dihydroxyphenyllactic acid.

### 2.2. XQG Ameliorated II/R Induced Intestinal Morphological Damage

**Figure 2:**
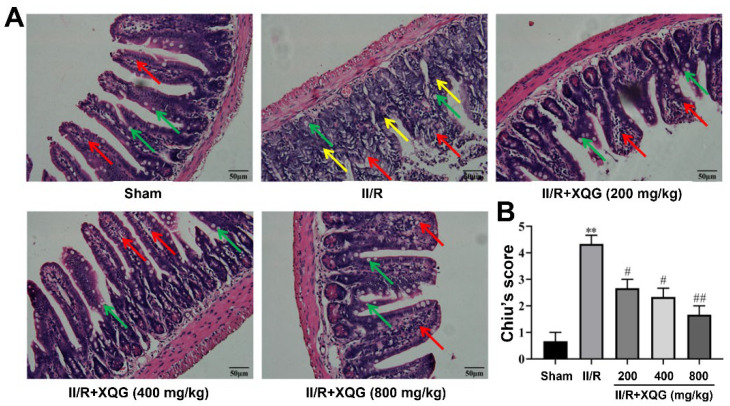
Protective effects of XQG against II/R-induced ileum morphological damage. (**A**) H&E staining of ileum histological lesion (scale = 50 μm). Goblet cells, lamina propria, and inflammatory cell infiltration were indicated by arrows with green, red and yellow colors, respectively; (**B**) Chiu’s score of ileum tissue. All values were expressed as mean ± SEM (*n* ≥ 3). ** *p* < 0.01 vs. sham group while ^#^ *p* < 0.05 and ^##^ *p* < 0.01 vs. II/R group.

Hematoxylin–eosin (H&E) staining showed that the intestinal mucosal epithelia in the sham group were intact in shape and neatly arranged in structure, with clearly visible goblet cells. Significant intestinal morphologic alterations, massive inflammatory cell infiltration, and lamina propria disintegration were observed in the intestinal mucosal epithelia in the II/R-injured mice. The intestinal morphologic damage was markedly improved by XQG (200, 400, and 800 mg/kg) pretreatment, with the intestinal histological injury scores (Chiu’s score) decreasing in a dose-dependent manner (Figure 2).

### 2.3. XQG Ameliorated II/R-Induced Intestinal Barrier Injury

**Figure 3:**
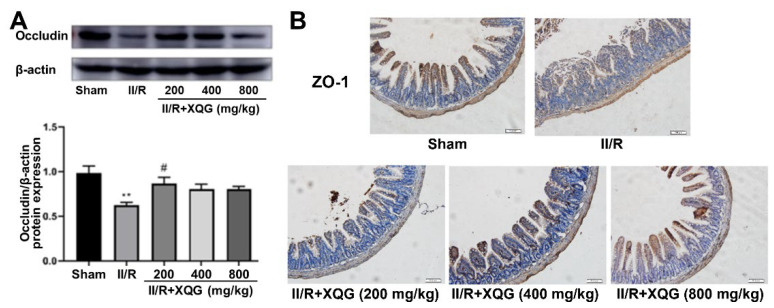
XQG alleviated II/R-induced intestinal barrier injury. (**A**) The expression level of Occludin in intestinal tissue; (**B**) Immunohistochemical expression of ZO-1 in intestinal tissue (scale = 100 μm). All values were expressed as mean ± SEM (*n* ≥ 3). ** *p* < 0.01 vs. sham group while ^#^ *p* < 0.05 vs. II/R group.

The degree of the intestinal epithelial barrier damage was assessed by examining the expression of the tight junction proteins ZO-1 and Occludin. Western blot and immunohistochemistry analysis showed that the tight junction proteins were significantly downregulated during the II/R injury, indicating the serious intestinal barrier damage, and the XQG pretreatment reversed the dysregulation of the tight junction proteins (Figure 3). The results showed that XQG pretreatment could alleviate II/R-induced intestinal barrier injury.

### 2.4. XQG Alleviated II/R-Induced Oxidative Stress and the Inflammatory Response

**Figure 4:**
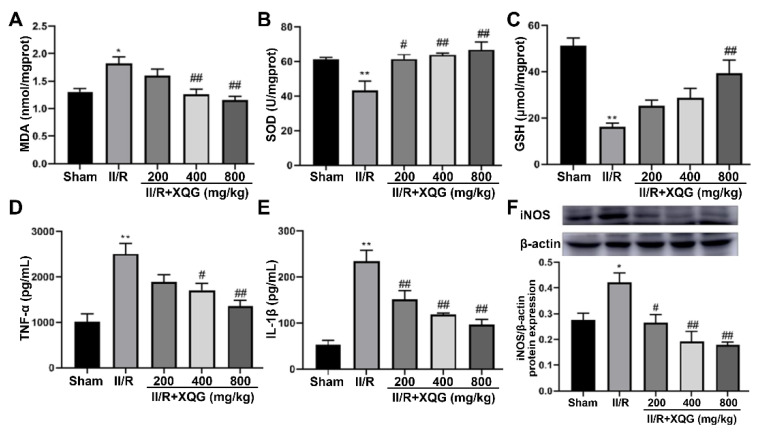
XQG alleviated oxidative stress and inflammatory response induced by II/R. Effects of XQG on (**A**) MDA; (**B**) SOD; (**C**) GSH; (**D**) TNF-α; (**E**) IL-1β; and (**F**) iNOS in intestinal tissues. All values were expressed as mean ± SEM (*n* ≥ 3). compared with sham group, * *p* < 0.05 and ** *p* < 0.01 vs. sham group, ^#^ *p* < 0.05 and ^##^ *p* < 0.01 vs. II/R group.

Excessive oxidative stress is one of the main nosogenesis of II/R injury. The oxidative stress injury was evaluated by measuring the expression levels of MDA (a biomarker of oxidative damage) and SOD and GSH (biomarkers of antioxidant capacity) in the intestinal tissue. The upregulation of MDA and downregulation of SOD and GSH were observed in the II/R-injured group. The XQG pretreatment reversed these dysregulations in a dose-dependent manner (Figure 4A–C). Further, our results showed that the levels of pro-inflammatory cytokines, including IL-1β and TNF-α, and the induced iNOS were increased in the II/R-injured mice compared with the sham group. The XQG pretreatment significantly reduced these increased proinflammatory cytokines (Figure 4D,E). The above results showed that XQG pretreatment could alleviate II/R-induced oxidative stress and inflammatory response.

### 2.5. XQG Alleviated II/R-Induced Cell Apoptosis

**Figure 5:**
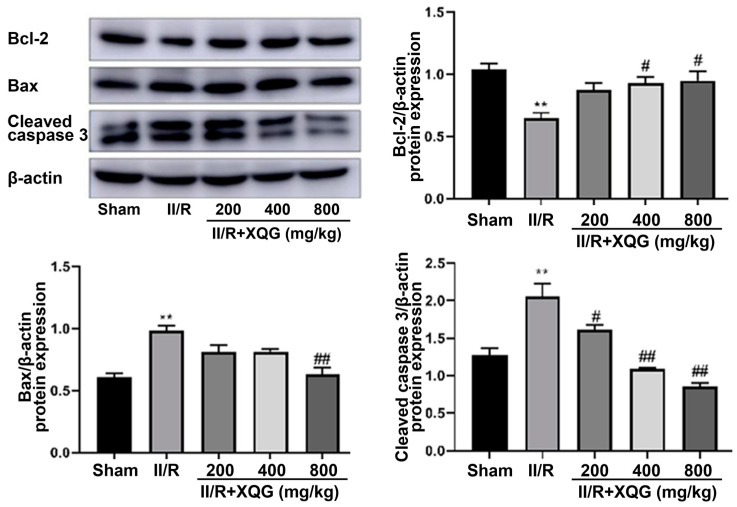
XQG alleviated II/R-induced apoptosis. Western Blot analysis of intestinal bcl-2, Bax, and cleaved caspase3. All values were expressed as mean ± SEM (*n* ≥ 3). ** *p* < 0.01 vs. sham group, *^#^ p <* 0.05 and *^##^ p <* 0.01 vs. II/R group.

Western blot analysis showed that the proapoptotic protein Bax and the apoptotic executive protein-cleaved caspase3 were upregulated, and the anti-apoptotic protein Bcl2 was downregulated upon II/R injury (Figure 5). Compared with the II/R group, the XQG pretreatment reversed the expression trends of the above apoptosis-related proteins. The results showed that the XQG pretreatment could reduce the apoptosis, induced by II/R.

### 2.6. XQG Pretreatment Alters Gene Expression in II/R Mice

**Figure 6:**
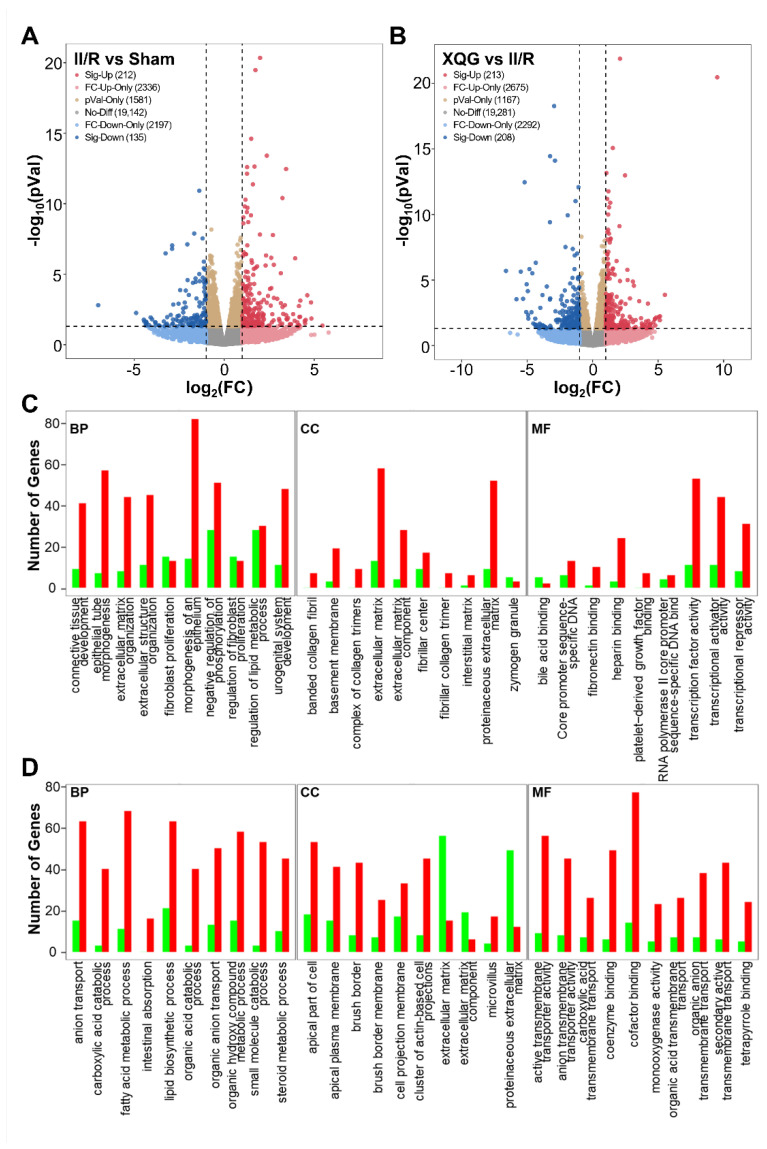
Differentially expressed genes and GO functional annotation. (**A**,**B**) Differentially expressed genes after II/R injury (**A**) and XQG treatment (**B**); (**C**,**D**) GO functional annotation of the differentially expressed genes after II/R injury (**C**) and XQG treatment (**D**).

To reveal the precise molecular mechanisms by which the XQG alleviated the II/R-induced injury in mice, mRNA-Seq whole-transcriptome analysis was used to examine the differentially expressed genes in the intestinal tissues of the II/R-injured mice and the XQG-treated II/R-injured mice. As a result, 347 differentially expressed genes (DEGs) were identified in the II/R group versus the sham group (two-fold change cutoff with *p* < 0.05), among which 212 genes were upregulated, and 135 genes were downregulated (Figure 6A). To further explore the biological significance of the differentially expressed proteins, the DEGs were categorized according to GO functional annotation terms (Figure 6C). The II/R-induced DEGs were mainly assigned to the activity of transcriptional regulation, including transcription factor activity, transcriptional activator activity, and transcriptional repressor activity, and participated in several biological processes relevant to the epithelial cell structure, including epithelial tube morphogenesis, extracellular structure organization, and the morphogenesis of an epithelium. Additionally, 421 genes were differentially expressed in the XQG-treated group as compared to the II/R group, with 213 genes upregulated and 208 genes downregulated (Figure 6B). Those DEGs were mainly located in membrane and actin-based cell projections clusters and participated in ion transport and metabolic processes, suggesting the restoration of intestinal function (Figure 6D).

### 2.7. XQG Alleviated II/R-Induced Injury via the PPARα/NF-κB Signaling Pathway

**Figure 7:**
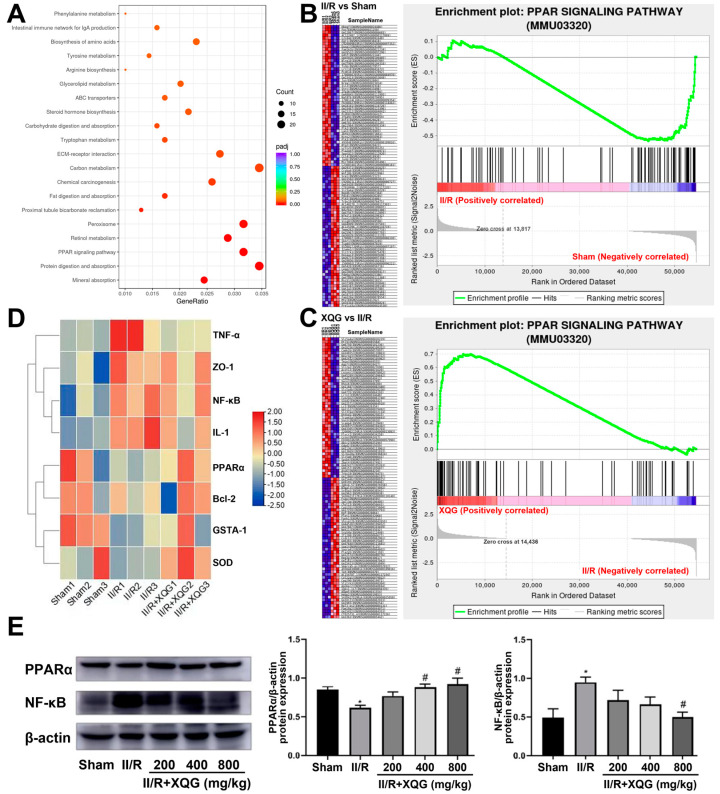
KEGG pathway enrichment. (**A**) KEGG enrichment result of DEGs after XQG administration; (**B**,**C**) Enrichment plots of PPAR signaling pathway from GSEA analysis after II/R injury (**B**) and XQG administration (**C**); (**D**) Heat map of gene expression levels of PPARα, NF-κB, Bcl2, TNF-α, IL-1, Gsta1 and SOD; (**E**) The protein expression levels of PPARα and NF-κB in the intestinal tissue. All values were expressed as mean ± SEM (*n* ≥ 3). * *p* < 0.05 vs. sham group, ^#^ *p* < 0.05 vs. II/R group.

To further investigate the XQG target and relative mechanism of action, a KEGG-based biological analysis of the DEGs differentially expressed upon the XQG administration was conducted. As shown in Figure 7A, the PPAR-signaling pathway was significantly enriched. The gene set enrichment analysis (GSEA) showed that the PPAR-signaling pathway was significantly suppressed in the II/R-injured group, and the XQG pretreatment restored the suppressed PPAR-signaling pathway (Figure 7B,C). Further, the gene expression levels of PPARα, NF-κB, anti-apoptotic protein Bcl2, inflammatory factor TNF-α and IL-1, and the antioxidant enzymes, Gsta1 and SOD, were extracted. As a result, the gene expression levels of PPARα, Bcl2, Gsta1, and SOD were decreased upon the II/R injury, while the gene expression levels of NF-κB, TNF-α, and IL-1 were increased, and XQG pretreatment significantly reversed the dysregulation of those genes (Figure 7D). The WB analysis further confirmed the restoration of the decreased PPARα and the downregulation of NF-κB after the XQG pretreatment (Figure 7E). The result confirmed the amelioration of XQG on the II/R-induced oxidative stress, inflammatory response, and cell apoptosis, and the regulation of the PPARα/NF-κB pathway.

## 3. Discussion

II/R injury is a surgical emergency, with surgical treatment as the only clinically feasible treatment and limited preventions. The II/R may lead to impaired intestinal barrier function with increased intestinal permeability and translocation of intestinal flora, resulting in severe local and systemic inflammation and multiple organ dysfunction syndrome. A large number of studies have shown that oxidative stress, inflammation, and cell death are the main pathogenesis of II/R [20,21]. Based on our previous studies, the phenolic acids’ extract of Xiqingguo (XQG for short), the unripe fruit of *Terminalia chebula* Retz., has strong anti-inflammatory, anti-oxidative, and anti-apoptotic effects. Therefore, we proposed that XQG could effectively ameliorate II/R injury.

The results of the morphological evaluation and biochemical determination supported our hypothesis. The intestinal barriers include the mechanical barriers, chemical barriers, immune barriers, and biological barriers. The mechanical barrier is a complete tight intercellular junction formed by the intestinal epithelial cells, the most important barrier of the intestinal mucosa. The tight intercellular junctions of the intestinal epithelial cells are composed of tight junction proteins, such as Occludin and ZO-1 [22]. The disruption of the intestinal barrier allows lumen microbes, endotoxins, and other toxins to enter the bloodstream and other organs. The destruction can lead to serious clinical consequences, such as sepsis, acute respiratory distress syndrome, and multiple organ failure, with a high rate of mortality [23]. Our study showed that the XQG pretreatment restored the II/R-induced downregulated expression levels of the tight junction protein Occludin and ZO-1, suggesting a protective effect of XQG on the II/R-induced intestinal barrier injury in mice.

The studies have shown that oxidative stress and inflammation are the main factors leading to II/R damage [24]. The reactive oxygen species (ROS) is a byproduct of normal cell metabolic activities; low or moderate ROS have beneficial effects on some of the physiological processes, but disproportionate ROS generation will damage the body’s homeostasis and lead to oxidative damage [25]. Ischemia/reperfusion (I/R) injury and various inflammatory processes can also lead to increased ROS levels. MDA is the final product of lipid peroxidation and reflects the state of the ROS levels, thus reflecting the state of oxidative stress [26]. SOD is an enzyme involved in protecting the cells from ROS damage and is used to evaluate the antioxidant level of tissues [27]. GSH is considered as a major mitochondrial antioxidant, and its loss significantly increases the sensitivity of mitochondrial structures to ROS-mediated damage [28]. In this study, we evaluated the content of MDA, SOD, and GSH in the intestinal tissues of each group, and the results showed that, compared with the sham group, the production of MDA in the II/R group increased, while the content of SOD and GSH decreased significantly. The XQG pre-administration restored the redox homeostasis in mice. Further, the XQG also reduced the expression levels of the pro-inflammatory cytokines (TNF-α, IL-1β, and iNOS). These results suggest the protective effects of XQG on II/R-induced oxidative stress injury and inflammation.

Previous studies have shown that apoptosis is the main mode of intestinal mucosal cell death after II/R injury [29]. Our results showed that XQG pre-administration increased the expression of Bcl-2 in intestinal tissues and decreased the expression of Bax and cleaved caspase 3, suggesting that XQG reversed the cell apoptosis in vivo by inhibiting the activated apoptosis-related proteins.

Transcriptomic studies were performed to investigate the relative mechanism of XQG protecting against II/R injury. The expression of the genes related to the intestinal barrier, inflammation, oxidative stress, and apoptosis were consistent with the above biochemical determinations. Further, the differentially expressed genes (DEGs) were well studied. In total, 347 DEGs were identified in the II/R group versus the sham group, which mainly participated in several biological processes relative to the epithelial cell structure, including epithelial tube morphogenesis, extracellular structure organization, and morphogenesis of an epithelium. Meanwhile, 421 DEGs were found in the XQG-treated group as compared to the II/R group, and those DEGs were mainly located in the membrane and actin-based cell projections’ clusters and participated in ion transport and metabolic processes, suggesting the restoration of intestinal function.

Peroxisome proliferator-activated receptor (PPAR) is a member of the nuclear receptor superfamily, consisting of PPAR-α, PPAR-β/δ, and PPAR-γ, and plays an important role in sugar and lipid metabolism as the ligand-induced transcription factor [30]. PPARα has shown strong anti-inflammatory ability and is reported to be involved in the regulation of cell apoptosis, lipid metabolism, and the inflammatory response [31,32]. Activated PPARα is found to inhibit inflammation through negatively interfering with the activities of the pro-inflammatory transcription factors (STAT), the activator protein 1 (AP-1), and the nuclear factor -κB (NF-κB), and therefore influences the production and release of inflammatory factors, such as TNF-α and IL-1β [33,34,35]. In addition to inflammation regulation, PPARα also participates in various pathophysiological processes, including oxidative stress, apoptosis, and even tumorigenesis and cancer progression [14]. The KEGG-based biological analysis of DEGs that were differentially expressed upon XQG administration were further investigated to explore the target and relative action mechanism of XQG, and the PPAR-signaling pathway was significantly enriched. Gene set enrichment analysis (GSEA) showed that the PPAR-signaling pathway was significantly suppressed in the II/R-injured group, and the XQG pretreatment restored the suppressed PPAR-signaling pathway. Both the gene and protein expression levels of PPARα and Bcl-2 were downregulated, while the NF-κB, TNF-α, IL-1β, ZO-1, SOD, and GSH were increased upon II/R injury. The XQG pretreatment restored the dysregulation of those genes. The above results suggest that XQG can activate PPARα and inhibit the NF-κB-signaling pathway, thereby inhibiting the II/R-induced inflammatory response, oxidative stress, and cell apoptosis.

## 4. Materials and Methods

### 4.1. Main Instruments and Reagents

The BX63 positive fluorescence microscope was obtained from OLYMPUS Company. The gel imager was obtained from GE Company. The MDA, SOD, and GSH detection kits were obtained from Nanjing Jiancheng Bioengineering Institute. The ELISA kits were acquired from Beyotime Biotechnology Co., Ltd. The primary antibody of PPARα (WL00978) was purchased from Wanlei Biotechnology Co., Ltd (Shenyang, China). The primary antibodies of NF-κB (66535-1-lg), iNOS (18985-1-AP), Occludin (27260-1-AP), Bax (50599-2-lg), Bcl-2 (26593-1-AP), β-actin (66009-1-Ig), and Cleaved caspase3 (19677-1-AP), the UltraPolymer Goat anti-Rabbit IgG (H&L)-HRP (PR30011), and the UltraPolymer Goat anti-Rat IgG (H&L)-HRP (PR30012) were all obtained from the Proteintech Group Inc. (Wuhan China). The primary antibody of ZO-1 (bs-1329R) was purchased from Bioss Biotechnology Co., Ltd. (Beijing, China). The multicolor pre-stained protein ladder marker was obtained from Shanghai Epizyme Biomedical Technology Co., Ltd. (3 μL/channel, 10 kDa-250 kDa, WJ102, Shanghai, China).

The Chinese herbal medicine, *Fructus Chebulae Immaturus,* was purchased from Beijing Tongrentang Pharmacy (Dalian Xi’an Road Branch, Dalian, China). The phenolic acids’ extraction was obtained according to the method provided in reference [18,36], with minor revisions. First, 500 g *Chebulae Fructus immaturus* was ground into powder and passed through a 30-mesh sieve before use. The powder was extracted by 75% ethanol (solid/liquid ratio was 1:25) at 80 °C for 3 h. Subsequently, the filtrates were concentrated by rotary evaporation at 60 °C under reduced pressure, then chromatographed on a D101 macroporous resin column, and subsequently eluted with 20%, 30%, 40%, 50%, and 60% ethanol. The fraction eluted by 60% ethanol was collected and lyophilized; finally, the obtained samples were stored at −20 °C for further experiments.

### 4.2. Animals

The SPF healthy C57BL/6 mice (male, 6–8 weeks old, 18–22 g weight) were purchased from the SPF Experimental Animal Center of Dalian Medical University (certificate: SCXK (Liao) 2020-0001). The mice were divided randomly into five groups with eight mice in each group as follows: (1) Sham group; (2) II/R group; (3) II/R + XQG 200 mg/kg group; (4) II/R + XQG 400 mg/kg group; (5) II/R + XQG 800 mg/kg group. Each mouse was given 0.4 mL 0.5% sodium carboxymethyl cellulose (CMC-NA) with different concentrations of XQG once a day by gavage for 5 days.

Before the establishment of the II/R injury model, the mice were fasted with free access to water for 12 h. The mice were anesthetized with 3% pentobarbital sodium (50 mg/kg) and fixed in the supine-face up position. We occluded the superior mesenteric artery with a microvascular clamp for 30 min, then removed the clamp for reperfusion for 2 h. After reperfusion, the mice were sacrificed by cervical dislocation. The ileum tissue was quickly removed and then fixed by paraformaldehyde or quickly frozen with liquid nitrogen for subsequent experiments. The ileum tissues of three mice in each group were selected randomly for the transcriptome sequencing analysis, and at least three of the remaining animals were randomly selected for the WB experiments and biochemical analysis.

### 4.3. Histopathological Examination

The isolated ileum segment was fixed in 4% paraformaldehyde for at least 24 h and further used for hematoxylin–eosin (H&E) staining after paraffin embedding. The pathological changes of the ileum were observed under a microscope and assessed by Chiu’s scale, which scores normal villi as 0, deformed villi tips and capillary hemorrhage as 1, moderate elevation of the epithelium as 2, severe progressive elevation of the epithelium as 3, disruption of villi, dilated capillaries, and loss of epithelium in the lamina propria as 4, and digestive disintegration, hemorrhage, and dilatation of the lamina propria as 5.

### 4.4. Biochemical Indicators Detection

The contents of MDA, SOD, GSH, TNF-α, and IL-1β in the intestinal tissue were detected according to the user instructions provided with the kits.

### 4.5. Immunohistochemical Analysis

The paraffin-embedded ileum sections were dewaxed, washed with PBS, added to 3% hydrogen peroxide solution, and incubated in a closed buffer solution for 30 min. The ileum sections were incubated with diluted ZO-1 antibody (1:500 dilution) overnight, then incubated with horseradish coupled with goat anti-rabbit antibody (HRP) and washed with PBS. Finally, DAB was used for color rendering, and a microscope was used for imaging.

### 4.6. Transcriptome Sequencing

The ileum tissues of three mice were selected randomly from the control group, model group, and the XQG high-dose extract group respectively. The samples were then sent to Dalian Laiwo Biomedical Technology Co., Ltd. (Dalian, China) for RNA sequencing analysis.

### 4.7. Western Blot

The protein samples were extracted from the ileum tissues by RIPA tissue cell lysate and quantified by the BCA method. The protein samples were separated by 10%, 12%, or 15% SDS-PAGE, according to the molecular weight of the protein, and then transferred to the PVDF membranes. Then, the membranes were blocked by 5% defatted milk for 2 h at 37 °C and incubated with the following primary antibodies: PPARα, NF-κB, iNOS, Occludin, Bax, Bcl-2, cleaved caspase3, and β-actin (all of the antibodies were diluted at a 1:1000 ratio). The PVDF membranes were washed with TBST three times and then incubated with an HRP-labeled secondary antibody (1:1000 dilution) at room temperature for 2 h. Finally, the membranes were exposed to the ECL detection system. The protein quantification was determined in optical density units using Image Lab software (Bio-Rad, CA, USA) and normalized to the corresponding sample protein expression of β-actin.

### 4.8. Statistical Analysis

We analyzed the experimental results by mean ± SEM and analyzed the data statistically by Excel. The data were compared between three or more groups using one-way ANOVA with a follow-up Tukey test for the statistical significance, with *p* < 0.05 considered as statistically different.

## 5. Conclusions

In conclusion, the XQG effectively alleviated the II/R-induced intestinal morphological damage and intestinal barrier injury by decreasing the oxidative stress, inflammatory response, and cell death though activating PPARα and inhibiting the NF-κB-signaling pathway. Our results suggest the potential usage of XQG as a new candidate to alleviate II/R injury.

## Data Availability

Not applicable.

## Supplementary Materials

The following supporting information can be downloaded at: https://www.mdpi.com/article/10.3390/molecules27165227/s1, Figure S1: The beak chromatographic base peak of XQG in HPLC-MS/MS analysis; Table S1: The components of XQG identified by HPLC-MS/MS analysis.

## Sample Availability

Samples of the XQG extracts are available from the authors.
