# Supplementary material for: Phenolic Acids from Fructus Chebulae Immaturus Alleviate Intestinal Ischemia-Reperfusion Injury in Mice through the PPARα/NF-κB Pathway
Source: Molecules. 2022 Aug 16;27(16):5227. doi: 10.3390/molecules27165227 (PMC9415796; doi:10.3390/molecules27165227)
Supplement: Supplementary file 1 [file molecules-27-05227-s001.zip › molecules-1807030-supplementary.pdf]

Phenolic acids from *Fructus Chebulae Immaturus* alleviate intestinal ischemia–reperfusion injury in mice through the PPARα/NF-κB pathway

Junjie Liu[1], Bin Li[2,3], Jing Liu[2,3*], Feng Qiu[1], Yunpeng Diao[2,3*], Yuxin Lei[2], Jianjun Liu[2], Wei Zhang[1*]

[1] Department of Pharmacy, First Affiliated Hospital of Dalian Medical University, Dalian, 116011, China
[2] College of Pharmacy, Dalian Medical University, Dalian, 116044, China
[3] Dalian Anti-Infective Traditional Chinese Medicine Development Engineering Technology Research Center, Dalian 116044, China.
* Correspondence: liujing8166@dmu.edu.cn (J.L.); diaoyp@dmu.edu.cn (Y.P.D.); zhangwei1@firsthosp-dmu.com (W.Z.).

Figure S1

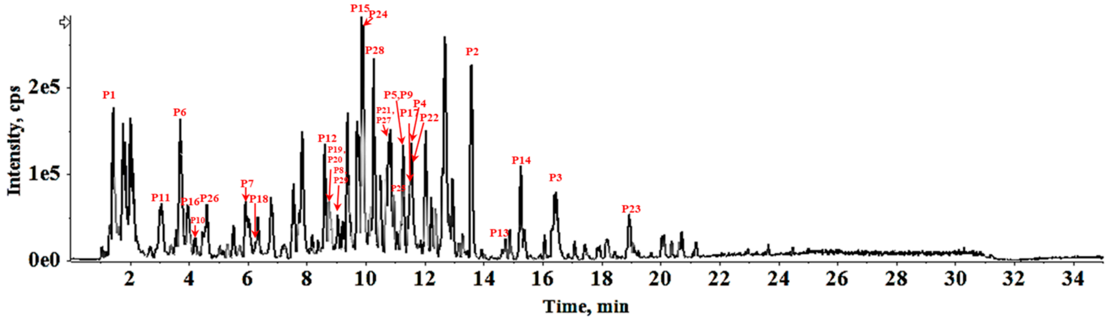

Figure S1 The beak chromatographic base peak of XQG in HPLC-MS/MS analysis.

Table S1

Table S1 The components of XQG identified by HPLC-MS/MS analysis.

ID	Component Name	Retention Time (min)	Formula	ESI-	cal m/z
P1	Shikimic acid	1.47	C7H10O5	[M-H]-	173.0455
P2	Chebuloside 2	13.57	C36H58O11	[M+Cl]-	701.3673
P3	Arjugenin	16.43	C30H48O6	[M-H]-	503.3370
P4	Ellagic Acid	11.53	C14H6O8	[M-H]-	300.9990
P5	Eschweilenol C	11.21	C20H16O12	[M-H]-	447.0559
P6	Gallic acid	3.70	C7H6O5	[M-H]-	169.0142
P7	Dihydroxybenzoic Acid	5.98	C7H6O4	[M-H]-	153.0191
P8	Gallic Acid Methyl Ester	9.03	C8H8O5	[M-H]-	183.0303
P9	3,4-Dihydroxyphenyllactic acid	11.28	C9H10O5	[M-H]-	197.0451
P10	Beta-Glucogallin isomer	4.16	C13H16O10	[M-H]-	331.0649
P11	Beta-Glucogallin isomer	2.99	C13H16O10	[M-H]-	331.0663
P12	Dehydrodigallic Acid	8.65	C14H10O10	[M-H]-	337.0200
P13	Dimethyl - ellagic acid	14.82	C16H10O8	[M-H]-	329.0303
P14	Chebuloside 1	15.25	C36H58O10	[M+Cl]-	685.3724
P15	Caffeic acid	9.75	C9H8O4	[M-H]-	179.0351
P16	Chebulic acid	3.95	C14H12O11	[M-H]-	355.0296
P17	Chebulinic acid	11.45	C41H32O27	[M-H]-	955.1028
P18	2,4-Dihydroxybenzoic acid	6.19	C7H6O4	[M-H]-	153.0194
P19	Punicalagin	8.74	C48H28O30	[M-H]-	1083.053
P20	Hamamelitannin	8.82	C20H20O14	[M-H]-	483.0751
P21	Chebulagic acid	10.80	C41H30O27	[M-H]-	953.0915
P22	Pentagalloylglucose	11.60	C41H32O26	[M-H]-	939.1102
P23	Arjunolic acid	18.93	C30H48O5	[M-H]-	487.3432
P24	Corilagin	9.86	C27H22O18	[M-H]-	633.0740
P25	a-D-Glucopyranose,1,2,3,4-tetrakis(3,4,5-trihydroxybenzoate)	11.05	C34H28O22	[M-H]-	787.1002
P26	Caftaric acid	4.46	C13H12O9	[M-H]-	311.0400
P27	Eugeniin	10.70	C41H30O26	[M-H]-	937.096
P28	Euphormisin M3	10.25	C27H24O18	[M-H]-	635.0854
P29	m-Galloylgallic acid	9.10	C14H10O9	[M-H]-	321.0253
